# Randomized controlled trial of robotic-assisted versus conventional laparoscopic fundoplication: 12 years follow-up

**DOI:** 10.1007/s00464-021-08969-y

**Published:** 2022-01-25

**Authors:** F. Lang, A. Huber, K. F. Kowalewski, H. G. Kenngott, F. Billmann, A. T. Billeter, L. Fischer, V. V. Bintintan, C. N. Gutt, B. P. Müller-Stich, F. Nickel

**Affiliations:** 1 Department of General, Visceral, and Transplantation Surgery, Heidelberg University Hospital, Im Neuenheimer Feld 420, 69120 Heidelberg, Germany; 2 Department of Urology, University Medical Center Mannheim, Heidelberg University, Theodor-Kutzer-Ufer 1-3, 68167 Mannheim, Germany; 3 Department of Surgery, Hospital Mittelbaden, Balgerstrasse 50, 76532 Baden-Baden, Germany; 4 Department of Surgery, University Hospital Cluj Napoca, Cluj Napoca, Romania; 5 Department of Surgery, Memmingen Hospital, Memmingen, Germany

**Keywords:** Laparoscopy, Nissen fundoplication, Robotic-assisted surgery, Quality of life, Symptomatic outcome, Treatment failure, Lundell score, Gastroesophageal reflux disease, Randomized controlled trial

## Abstract

**Aims:**

Numerous reports have addressed the feasibility and safety of robotic-assisted (RALF) and conventional laparoscopic fundoplication (CLF). Long-term follow-up after direct comparison of these two minimally invasive approaches is scarce. The aim of the present study was to assess long-term disease-specific symptoms and quality of life (QOL) in patients with gastroesophageal reflux disease (GERD) treated with RALF or CLF after 12 years in the randomized ROLAF trial.

**Methods:**

In the ROLAF trial 40 patients with GERD were randomized to RALF (*n* = 20) or CLF (*n* = 20) between August 2004 and December 2005. At 12 years after surgery, all patients were invited to complete the standardized Gastrointestinal Symptom Rating Scale (GSRS) and the Quality of Life in Reflux and Dyspepsia questionnaire (QOLRAD). Failure of treatment was assessed according to Lundell score.

**Results:**

The GSRS score was similar for RALF (*n* = 15) and CLF (*n* = 15) at 12 years´ follow-up (2.1 ± 0.7 vs. 2.2 ± 1.3, *p* = 0.740). There was no difference in QOLRAD score (RALF 6.4 ± 1.2; CLF 6.4 ± 1.5, *p* = 0.656) and the QOLRAD score sub items. Long-term failure of treatment according to the definition by Lundell was not different between RALF and CLF [46% (6/13) vs. 33% (4/12), *p* = 0.806].

**Conclusion:**

In accordance with previous short-term outcome studies, the long-term results 12 years after surgery showed no difference between RALF and CLF regarding postoperative symptoms, QOL and failure of treatment. Relief of symptoms and patient satisfaction were high after both procedures on the long-term. Registration number: DRKS00014690 (https://www.drks.de).

After introduction of laparoscopic techniques during the twentieth century, complex open surgical procedures such as esophageal reflux surgery were also performed as minimally invasive interventions with good outcomes and patient satisfaction. This applies to both short- and long-term follow-up, including objective evidence of reflux symptoms relief and medication dependence [1–3].

The introduction of robotic-assisted surgery systems potentially enables surgeons to transfer their skills from open surgery to minimally invasive procedures more easily compared to conventional laparoscopy despite immanent learning curves [4, 5]. The role of robotic-assisted surgery is especially promising for more complex procedures involving fine dissection in narrow spaces and suturing and knot tying such as in fundoplication for gastroesophageal reflux disease (GERD) [5–7]. After the first robotic-assisted Nissen fundoplication (RALF) reported by Cadiere in 1999 [8] it has been debated if the robotic approach could bring additional benefits for surgical treatment of GERD compared with the conventional laparoscopic fundoplication (CLF). Therefore, the aim of the randomized controlled ROLAF trial performed in our department in 2004–2005 was to investigate whether potential advantages of RALF are reflected in the postoperative outcome of patients such as is the case for other fields of surgery [9]. In the ROLAF trial RALF and CLF yielded similar both objective and subjective short-term and mid-term results apart from a shorter total operative time for RALF [10, 11]. Since GERD is a benign disease than can considerably impair the quality of life (QOL) of patients, the goal of treatment is to provide long-term relief of symptoms and improvement of QOL. The aim of the current study was therefore to evaluate predominantly the subjective long-term results of the randomized ROLAF trial more than a decade after surgery with a focus on QOL and reflux specific symptoms.

## Materials and methods

In the randomized ROLAF trial a total of 40 patients were operated in 2004–2005 in a single center. At the time of surgery all patients were over 18 years of age and had a history of GERD requiring an acid suppressive therapy with proton pump inhibitor (PPI) for at least 3 months during the preceding year. GERD was initially diagnosed by the presence of endoscopic esophagitis or by severe clinical symptoms which resolved with PPI therapy (positive PPI test) and was confirmed by gastrointestinal endoscopy, barium swallow and 24-h pH monitoring. Exclusion criteria were previous major upper abdominal surgery, obesity with a body mass index (BMI) exceeding 40 kg/m^2^ and evidence of primary oesophageal disorders such as achalasia, sclerodermia or malignant diseases. Eligible patients were randomly assigned to either RALF or CLF the day before surgery. All CLF procedures were carried out by surgeons who had previously performed more than 30 CLF procedures and thus had completed their learning curve [12]. Both CLF and RALF were performed with a Nissen fundoplication. Details of the operative procedure have been published earlier [11]. RALF was conducted by a single surgeon after he had done a total of 30 RALF procedures and was experienced in robotic-assisted laparoscopic procedures [4, 13] using the daVinci™ Surgical System (Intuitive Surgical, Sunnyvale, CA, USA). In the literature at the time the original ROLAF trial was conducted, a range of 20–30 procedures was considered to be the necessary number that a robotic surgeon must have completed to be considered experienced [14]. CLF was performed by three different surgeons using the same operative technique, including the surgeon conducting RALF. Study bias due to the fact that RALF was only performed by one surgeon as opposed to a total of three surgeons who performed CLF cannot be excluded. This bias was however minimized by the fact that all participating surgeons were highly experienced in the procedures performed by them as part of the study and by high standardization of the surgical technique. Early and mid-term results of the ROLAF trial have been published before [10, 11].

**Fig. 1:**
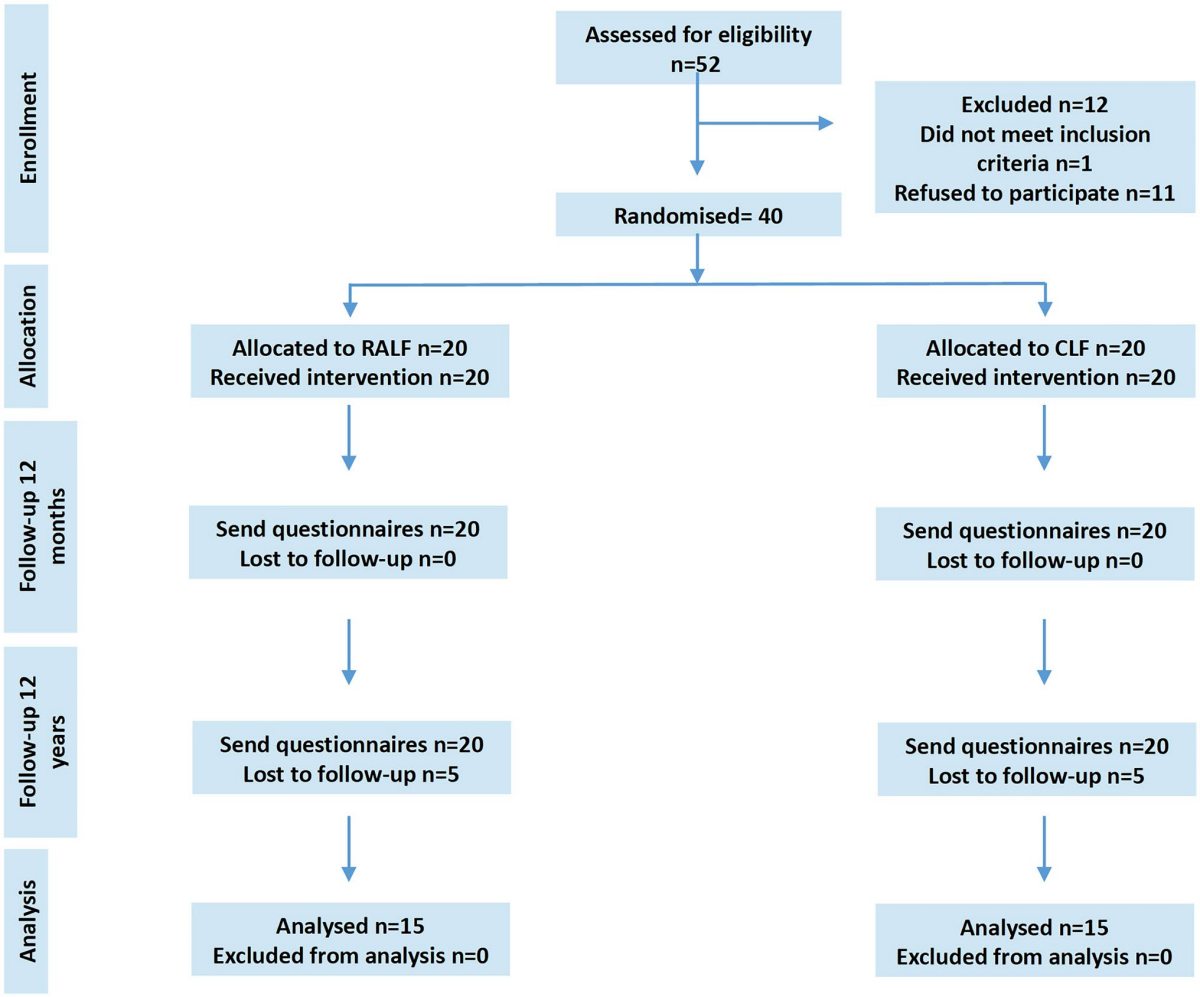
CONSORT Flowchart of the study including previously published results from the ROLAF trial

All patients included in the ROLAF trial were contacted for follow-up at least 12 years after surgery by mail and telephone (Fig. 1). In the very heterogeneous literature, a mean long-term follow-up of at least 10 years [15–19] and moreover is reported and defined for the assessment of quality of life in GERD, so it seemed reasonable and feasible to us, and we decided on a 12-year follow-up for organizational reasons. Patients were sent a standardized questionnaire and were offered a follow-up visit at the outpatient clinic or an assessment via telephone call. Symptomatic outcome and quality of life were determined by general health questionnaires. A standardized questionnaire on the psychometric characteristics of GERD-related symptoms recorded by the Gastrointestinal Symptom Rating Scale (GSRS) was used [20], a 15-item questionnaire that quantifies common GI symptoms such as abdominal pain, reflux, indigestion, diarrhea and constipation on a 7-point Likert scale (1 represents absence of bothersome symptoms). Patients were also asked to fill in the disease-specific questionnaire on quality of life in reflux and dyspepsia (QOLRAD). This is a disease-specific questionnaire on quality of life that focuses on the health concerns of people with GERD or dyspepsia [21, 22]. All its 25 questions are based on five domains that are important to patients: emotional stress, sleep disorders, eating and drinking problems, physical/social functions and vitality, measured on a scale of 1 (very much) to 7 (not at all). The lower the value, the more severe the impact on daily functioning.

Additionally, it was determined whether the patients experienced treatment failure 12 years after surgery, which was defined according to Lundell et al. [23, 24] as the occurrence of at least one of the following criteria: moderate or severe heartburn or acid regurgitation during the previous 7 days before the survey date, oesophagitis reflux subscore ≥ 3, esophagitis grade ≥ B, dysphagia value > 2 in combination with acid regurgitation value > 1, requirement for daily PPI treatment or need for reoperation due to recurrent GERD. Patients were all contacted personally and asked if they had received any EGD or reoperation at all since mid-term follow-up. Patients were asked to send the written findings to the authors if an EGD or reoperation or re-intervention had been performed. The most recent EGD was taken into account.

### Ethical considerations

The study protocol was approved by the institutional ethics committee at Heidelberg Faculty of Medicine (S-189/2018) and informed consent was obtained from every patient.

### Statistical analysis

All continuous data are presented as mean values with standard deviation and were compared using Mann–Whitney *U* test to determine the differences between the groups. Within group differences from baseline to follow-up were calculated using the paired *t* test. Group comparisons of dichotomized parameters were performed by Chi-squared tests. A double-sided p value of < 0.05 was considered statistically significant. All calculations were carried out using R version 3.5.2 (R project, R Foundation for Statistical Computing).

## Results

A total of 52 patients were screened for the original ROLAF trial according to the study protocol. Of these, 12 patients were not included because 11 patients refused to participate in the study one patient had a BMI of more than 40 kg/m^2^. 40 patients were enrolled in the study and received either RALF (*n* = 20) or CLF (*n* = 20). All of them were now contacted again for long-term follow-up at least 12 years after surgery—and were given the questionnaires. A total of 30 (RALF = 15, CLF = 15) replied (Fig. 1) and answered the questionnaires. Regarding the total of 10 patients who could not be included in the 12-year follow-up study, one patient died, two other patients wanted to be excluded from the study, and seven other patients could not be contacted due to change of residence although considerable efforts were made to reach all patients. From 25 (62.5%) patients the treatment failure could be assessed via outpatient visit of telephone interview. As previously published in detail, the preoperative patient characteristics of the two cohorts did not differ except for shorter operative time for RALF [11]. GSRS and QOLRAD scores within the first 12 months did not differ between the two procedures [10].

**Fig. 2:**
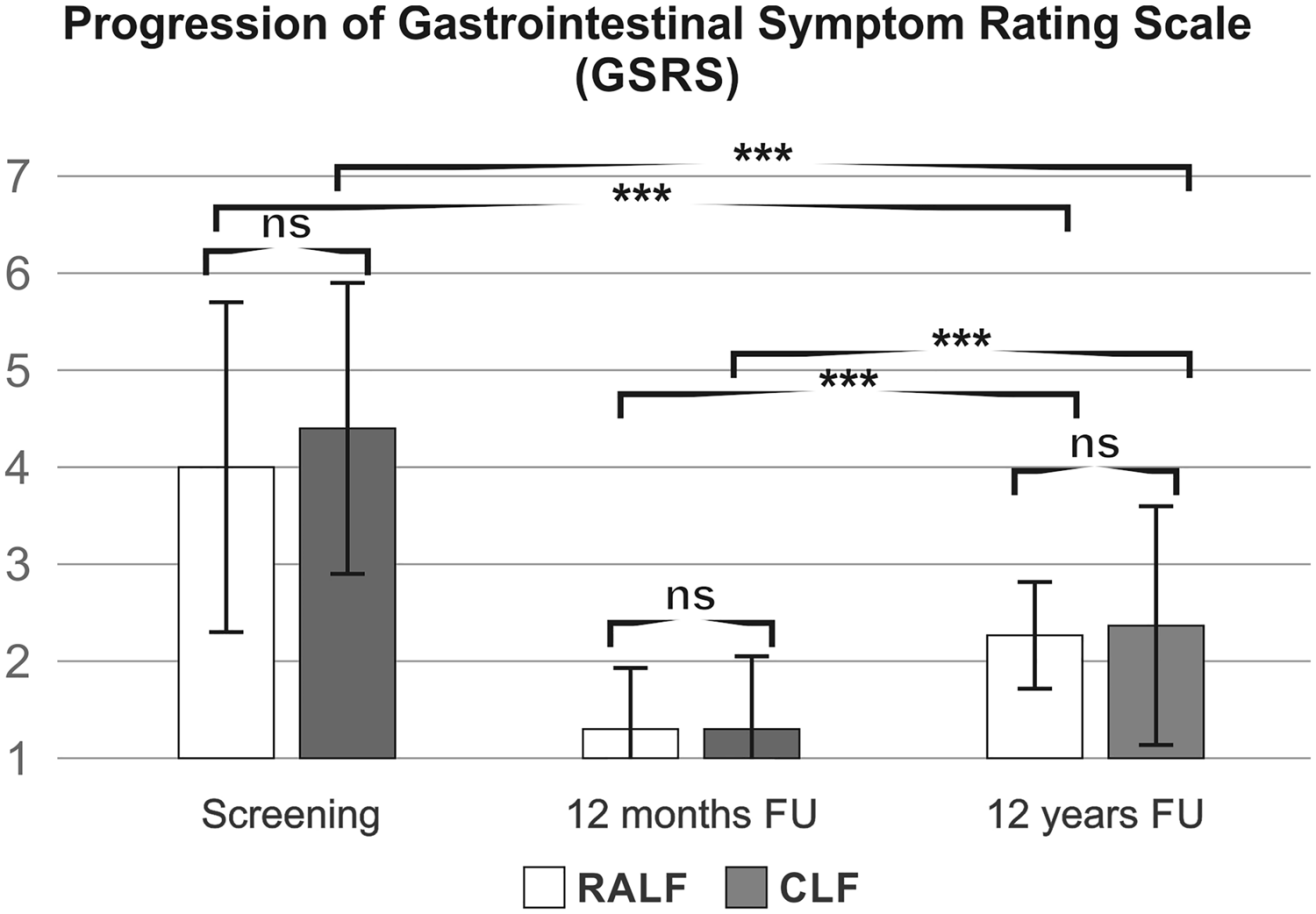
Progression of GSRS reflux scale. *RALF* robotic-assisted laparoscopic fundoplication, *CLF* conventional laparoscopic fundoplication, *GSRS* gastrointestinal symptom rating scale, 15-item questionnaire that quantifies common GI symptoms such as abdominal pain, reflux, indigestion, diarrhea and constipation on a 7-point Likert scale, 1 represents absence of bothersome symptoms, ns non-significant, mean ± SD, ***p < 0.001 [*t* -test between groups, mean ± SD, follow-up (FU)]

**Fig. 3:**
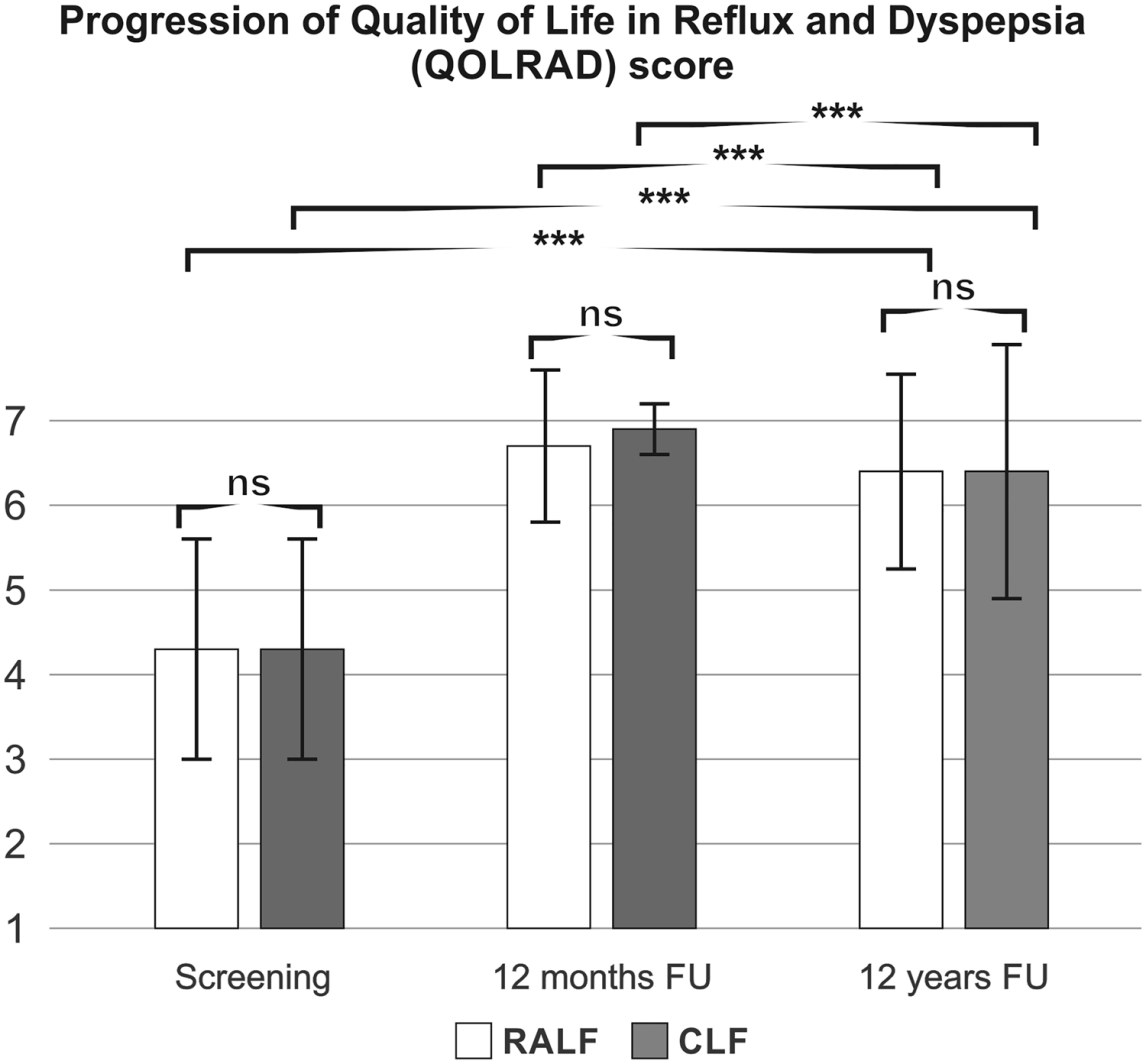
Progression of a QOLRAD score. *RALF* robotic-assisted laparoscopic fundoplication, *CLF* conventional laparoscopic fundoplication, *QOLRAD* Quality of life in reflux and dyspepsia, 25 questions based on five areas: emotional stress, sleep disorders, eating and drinking problems, physical/social functions and vitality, measured on a scale of 1 (very much) to 7 (not at all), ns non-significant, **p < 0.005, ***p < 0.001 [*t* test between groups, mean ± SD, follow-up (FU)]

At 12 years’ follow-up, the mean GSRS score was not different between RALF and CLF groups (Fig. 2). Accordingly, the mean GSRS reflux symptom score (heartburn and acid regurgitation) was similar between the groups (RALF: 1.6 ± 1.1, CLF 1.7 ± 1.4, *p* = 0.818). Two patients in the RALF group versus three patients in the CLF group had abdominal pain symptoms above the cut‐off values of 3 for reflux syndrome in the GSRS. Looking at the mean GSRS score specific for indigestion, there was no difference between CLF and RALF in long-term follow-up (RALF: 2.7 ± 1.1, CLF 2.7 ± 1.7, *p* = 0.975). However, 73% (11/15) in both groups reported indigestion above a cut-off value of 3, with fairly severe to very severe symptoms reported equivalently by only 33% (5/15) in both groups. The overall QOLRAD scores were good for both RALF and CLF and were significantly better than preoperatively (Fig. 3). Similarly, gastrointestinal symptoms on the GSRS Scale were less pronounced for both for RALF and CLF after 12 years compared to preoperative values (Fig. 2). However, compared to the 12-month follow-up data both the GSRS score and the QOLRAD scores were less favourable at the 12 years postoperative evaluation (Figs. 2 and 3).

When comparing the results of the two groups at 12 years’ follow-up in the overall QOLRAD score in all five dimensions, there was no significant difference (Table 1). A total of 13 percent in the RALF group (2/15) and 7 percent in the CLF group (1/15) recorded that they sometimes or permanently had a reduction in quality of life in terms of emotional stress, sleep disorders, eating and drinking problems, physical/social functions and vitality.

**Table 1:** Quality of life in reflux and dyspepsia (QOLRAD) at 12 years follow-up after surgery

	RALF (*n* = 15)	CLF (*n* = 15)	*p*
Emotional distress	6.4 ± 1.4 (1.2–7.0)	6.5 ± 1.6 (1.0–7.0)	0.288
Food/drink problems	6.5 ± 0.9 (3.5–7.0)	6.3 ± 1.6 (1.0–7.0)	0.867
Physical/social functioning	6.6 ± 1.0 (2.8–7.0)	6.4 ± 1.6 (1.0–7.0)	0.540
Sleep disturbance	6.4 ± 1.3 (2.2–7.0)	6.5 ± 1.5 (1.0–7.0)	0.939
Vitality	6.3 ± 1.4 (1.3–7.0)	6.3 ± 1.6 (1.0–7.0)	0.791

QOLRAD score at 12 years’ follow-up, 25 questions based on five areas: emotional stress, sleep disorders, eating and drinking problems, physical/social functions and vitality, measured on a scale of 1 (very much) to 7 (not at all), mean ± SD (range)

*RALF* robotic-assisted laparoscopic fundoplication, *CLF* conventional laparoscopic fundoplication, *QOLRAD* Quality of Life in Reflux and Dyspepsia

Failure of treatment according to the definition by Lundell was not different between RALF [46%, (6/13)] and CLF [33%, (4/12), *p* = 0.8063]. The signs of reflux recurrence are detailed in Table 2. The study population includes two patients with a BMI above 35 at the time of primary surgery. Both patients showed no treatment failure. Due to the rather small sample size a subgroup analysis was not possible.

**Table 2:** Signs of treatment failure according to Lundell et al. [23]

	RALF (n = 13)	CLF (*n* = 12)
Signs of treatment failure according to Lundell et al. [23]		
Esophagitis ≥ LA-B	1 (8%)	1 (8%)
Reoperation for reflux	0 (0%)	0 (0%)
GSRS reflux score ≥ 3	3 (25%)	2 (17%)
Daily PPI for reflux	4 (31%)	4 (33%)
Dysphagia combined with reflux score ≥ 2	1 (8%)	1 (8%)

Treatment failure according to Lundell et al. 23. Values denote numbers (%) of patients

*RALF* robotic-assisted laparoscopic fundoplication, *CLF* conventional laparoscopic fundoplication

## Discussion

In the present 12-year follow-up of the randomized controlled ROLAF trial there was no difference in QOL and reflux specific symptoms heartburn and acid regurgitation between RALF and CLF. The gastrointestinal quality of life and reflux associated symptoms at 12 years postoperatively were comparably good for RALF and CLF and were significantly better than preoperatively. The treatment efficacy also remained similarly stable in the 12-year follow-up for both RALF and CLF.

In the present study there was no difference between RALF or CLF for mid-term QOL and long-term QOL up to 12 years after surgery, in line with data from the literature which did not show any difference between RALF and CLF in improvement of quality of life and self-rated change in reflux symptoms at 3-, 6- and 12-month after surgery [13, 25] and 4 years [26] after surgery. However, the existing data are scarce, especially focusing on RALF versus CLF in terms of long-term quality of life. To date the results of a follow-up of over 30 years after open surgery is available for quality of life (QOL) [27], although the first open fundoplication performed by Nissen was as early as 1956 [28]. In general, antireflux surgery has been shown to improve QOL of patients with GERD, and it is common practice to evaluate disease-specific and general QOL [29]. For patients with GERD, the preoperative general QOL has been shown considerably lower than that expected for a healthy population [30]. However, in a study by Blomqvist et al., the general QOL of patients at 6 and 12 months after antireflux surgery has shown similar values compared with the normal population [31]. In addition to questionnaires on general QOL, disease-specific instruments are used to measure those areas of QOL that are specific to a particular disease. The longest follow-up series for QOL after CLF are currently limited to 20 years [16, 19, 32, 33]. Moreover, there is a lack of comparable long-term follow-up data in the literature regarding RALF. The current long-term follow-up study therefore adds evidence to the existing literature. The mean GSRS score describing overall discomfort of the patients in the present study 12 years after surgery related to reflux-specific symptoms was comparable to a healthy control population proving the efficacy of both CLF and RALF [16]. The long-term follow-up data based on the disease-specific questionnaire QOLRAD with particular focus on emotional distress, sleep disturbance, eating and drinking issues, physical and social functioning and vitality, were slightly inferior in the present study than the mean value of the healthy population, but in line with the comparable literature references for patients after antireflux surgery [34–36]. Although the excellent results obtained 12 months postoperatively in the ROLAF trial could not be completely maintained at 12 years, the GSRS values remained significantly better than preoperatively in line with the data present in the literature [27, 30, 31, 33]. It must be noted that antireflux surgery brought a durable improvement of QOL and reflux symptoms on the long-term in most available studies as also in the present study [10].

In the 12-year follow-up, both RALF and CLF showed objectifiable stable symptom control of GERD and low rates of treatment failure according to the Lundell definition [23, 24]. Parameters for the effectiveness of the treatment, such as requirement for daily PPI treatment, need for reoperation due to recurrent GERD or oesophagogastroscopy results, were queried on the basis of the patient assessment. In particular, treatment failure in patients with GERD is associated with lower life satisfaction, which has already been shown for treatment failure with PPIs [37]. On the other hand, previous studies have also associated psychological stress and poor quality of life with increased symptom severity in GERD patients [38]. Therefore, the long-term improvement of QOL is one of the main goals in the treatment of the majority of GERD patients and is actually more relevant for their well-being than the status of the objectively assessed parameters which often show a limited correlation with patients’ functional status, and satisfaction with therapy. Quality of life encompasses very diverse facets of human well-being with physical, emotional and social functioning. According to the WHO definition of health as "a state of complete physical, mental and social well-being and not merely the absence of disease or infirmity", quality of life is related to health and should be considered one of the most important postulates of modern medical science [39]. With its help, one can assess not only the physical but also the mental health of a person. In addition to the annually increasing number of publications focusing on quality of life in relation to GERD, the connection between objective tests such as pH impedance testing and quality of life has already been established [40] and thereby continues to increase the focus on the subjective and objective results, so that they can be compared more transparently.

Looking at the short-term results revealed by the ROLAF study, the minimally invasive antireflux surgery provided effective relief of symptoms and cure of erosive esophagitis. For the long-term follow-up, the results in the present study indicate a comparably stable efficacy of RALF and CLF and most of the patients reporting an overall QOL similar to that of the general population.

The present study has the advantage of re-evaluating two groups of patients within the framework of a randomized controlled trial with a long-term follow-up of 12 years. Although considerable efforts were made to reach all patients who had participated in the original ROLAF study, the 75% follow-up rate at 12 years after surgery is a limitation. Another limitation of the present study is the lack of objectively measured results. The two questionnaires used for the follow-up focus solely on the subjective evaluation of symptoms experienced by the patients and do not include objective parameters such as 24 h pH monitoring. In order to gain an impression of the treatment success of antireflux surgery over the last 12 years, the already established parameter of treatment failure according to Lundell was included as a measure. On the one hand, objective outcome parameters are included, such as a necessary reoperation or oesophagitis at least grade 2, but on the other hand, subjective parameters are also recorded here, such as the occurrence of moderate or severe heartburn or acid regurgitation during the previous 7 days before a hospital visit. Also, the question of whether PPI treatment was necessary daily for more than 8 weeks after antireflux surgery to control reflux symptoms was based on patient assessment and could be correlated and objectified only in patients who underwent EGD on their own initiative. Parameters for the effectiveness of the treatment, such as requirement for daily PPI treatment, need for reoperation due to recurrent GERD or oesophagogastroscopy results, were queried on the basis of the patient assessment. All results presented refer to the majority of patients with GERD. Special patient groups, such as patients in whom reflux contributes to lung graft failure or patients with possible progression of Barretts with dysplasia, and possible progression of other related diseases are excluded.

Since the subjective improvement of QOL and resolution of symptoms are the main goals of antireflux surgery the results of the present study are nonetheless relevant.

## Conclusion

In summary the present 12 years follow-up of the randomized ROLAF trial found no differences in treatment success between RALF and CLF. QOL and disease-specific symptoms were considerably improved at 12 years follow-up compared to the preoperative values, although there was a decline compared to short-term follow-up. In conclusion both RALF and CLF provide durable treatment of GERD. Both minimally invasive approaches were shown to be safe alternatives for the experienced surgeon. After the early results of the ROLAF trial, the authors no longer routinely used the robotic system in laparoscopic fundoplication since there were no significant advantages but there is the downside of higher cost for the robotic system compared to conventional laparoscopy. The authors of the current manuscript currently perform other upper gastrointestinal, pancreatic and colorectal procedures with the newer version of the robotic systems and are considering to restart performing hiatal procedures as part of a training pathway for a robotic program.
